# Salmonella Subpopulations Identified from Human Specimens Express Heterogenous Phenotypes That Are Relevant to Clinical Diagnosis

**DOI:** 10.1128/spectrum.01679-22

**Published:** 2022-12-12

**Authors:** Yismashoa Gebremichael, John Crandall, Rituparna Mukhopadhyay, Fengfeng Xu

**Affiliations:** a Microbial Diseases Laboratory, California Department of Public Health, Richmond, California, USA; Keck School of Medicine of the University of Southern California

**Keywords:** *Salmonella*, subpopulations, different phenotypes, genome analysis, clinical and public health interventions

## Abstract

Clonal bacterial cells can give rise to functionally heterogeneous subpopulations. This diversification is considered an adaptation strategy that has been demonstrated for several bacterial species, including Salmonella enterica serovar Typhimurium. In previous studies on mouse models infected orally with pure Salmonella cultures, derived bacterial cells collected from animal tissues were found to express heterogenous phenotypes. Here, we show mixed Salmonella populations, apparently derived from the same progenitor, present in human specimens collected at a single disease time point, and in a long-term-infected patient, these Salmonella were no longer expressing surface-exposed antigen epitopes by isolates collected at earlier days of the disease. The subpopulations express different phenotypes related to cell surface antigen expression, motility, biofilm formation, biochemical metabolism, and antibiotic resistance, which can all contribute to pathogenicity. Some of the phenotypes correlate with single nucleotide polymorphisms or other sequence changes in bacterial genomes. These genetic variations can alter synthesis of cell membrane-associated molecules such as lipopolysaccharides and lipoproteins, leading to changes in bacterial surface structure and function. This study demonstrates the limitation of Salmonella diagnostic methods that are based on a single-cell population which may not represent the heterogenous bacterial community in infected humans.

**IMPORTANCE** In animal model systems, heterogenous Salmonella phenotypes were found previously to regulate bacterial infections. We describe in this communication that different Salmonella phenotypes also exist in infected humans at a single disease time point and that their phenotypic and molecular traits are associated with different aspects of pathogenicity. Notably, variation in genes encoding antibiotic resistance and two-component systems were observed from the subpopulations of a patient suffering from persistent salmonellosis. Therefore, clinical and public health interventions of the disease that are based on diagnosis of a single-cell population may miss other subpopulations that can cause residual human infections.

## INTRODUCTION

Salmonella is a major cause of foodborne disease-related hospitalizations and deaths worldwide (1). While most salmonellosis manifests as a limited gastrointestinal tract infection causing diarrhea that does not need medical care, systemic infections may be difficult to treat because of the increasing prevalence of drug-resistant Salmonella (2–4). The vaccines developed for specific Salmonella serotypes or serovars are not broadly efficacious (5, 6). Though culture-independent tests can rapidly detect Salmonella DNA in patient specimens, culture isolation is necessary for phenotypic and genotypic characterization (7, 8). Typing work is usually performed by public health or reference laboratories and is critical for bacterial pathogenicity characterization, outbreak tracing, and disease control. Serotyping methods have been traditionally used to profile the bacterial surface exposed antigens of O (lipopolysaccharides [LPS]), H (flagella), and Vi (capsular polysaccharides), which are made variable by Salmonella to evade host immune response. Molecular methods to resolve genetic differences between Salmonella isolates that share the same serotypes include pulsed-field gel electrophoresis (PFGE), multilocus sequence typing (MLST) of several housekeeping genes, and, more recently, whole-genome sequencing (WGS). WGS significantly increases genotypic resolution by scanning the entire genome for single nucleotide polymorphisms (SNPs) or allele variations in thousands of genes (i.e., whole-genome MLST) (9, 10). SNP analysis has been used to establish a detailed phylogeny among isolates from different patients (11, 12), as well as among longitudinal isolates from the same patient persistently infected with Salmonella enterica serovar Enteritidis (13) or Salmonella enterica serovar Typhimurium (14, 15). Typically, the typing results obtained by the aforementioned methods from one single-colony cell type or isolate have been determined for each Salmonella human infection. Detection of heterogenous Salmonella subpopulations is technically difficult with the current test systems for identification and antibiotic susceptibility testing and is routinely not performed by clinical laboratories. During routine serotyping of clinical Salmonella isolates submitted to our laboratory, we observed mixed Salmonella cell populations in some specimens.

## RESULTS AND DISCUSSION

**FIG 1:**
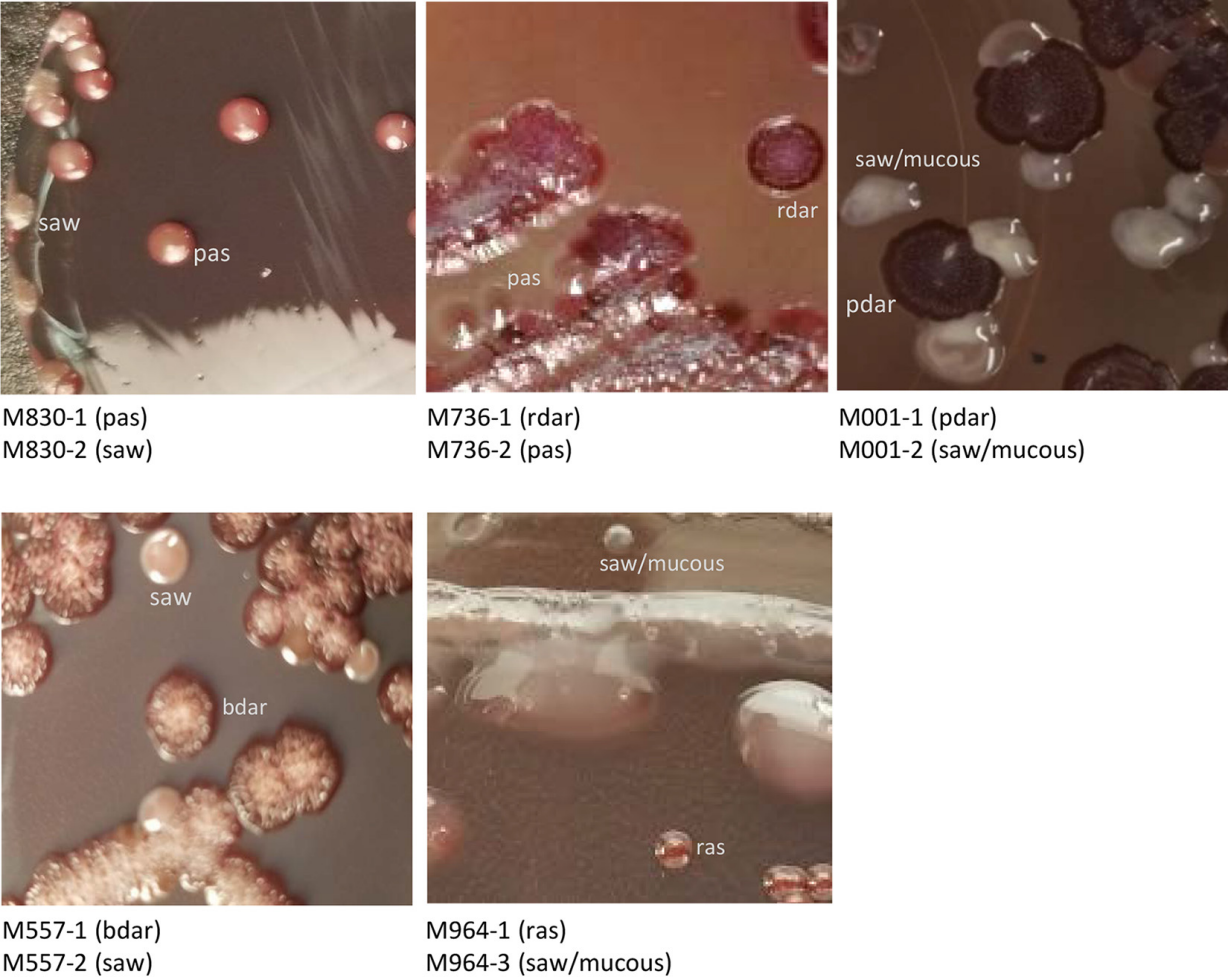
Salmonella bacteria from a clinical specimen express heterogeneous morphotypes on Congo red agar. Specimens M830, M736, M001, M557, and M964 were each shown to contain colonies of two distinctive morphotypes. Their appearance and associated abbreviated descriptions related to bacterial curli and cellulose expression are (i) red, dry, and rough (rdar; high expression of curli and cellulose); (ii) brown, dry, and rough (bdar; high expression of curli, but not cellulose); (iii) pink, dry, and rough (pdar; high expression of cellulose, but no curli); (iv) smooth and white (saw; no expression of curli or cellulose); (v) red and smooth (ras; low expression of curli and cellulose); and (vi) pink and smooth (pas; low expression of cellulose). Expression of curli and cellulose was well recognized previously to impact biofilm formation by bacteria (16, 17). All cultures were incubated at 35°C for 48 h.

As part of an internal study reviewing two different serotypes obtained from one specimen, serotyping was performed on 10 agar-plated colony cells. We detected more than one serotype associated with individual colony picks. Two such stool specimens, M211 and M378, and their carried isolates of heterogenous serotypes are described here (Table 1). We also observed heterogeneous Salmonella from serologically nontypeable urine specimens, M830, M736, M001, M557, and M964 (Table 1). These specimens were each shown on Congo red (CR) agar to carry distinctive colony morphotypes (Fig. 1), indicative of specific biofilm formation ability by bacteria (16, 17). M964 was initially observed to have mucoid and nonmucoid forms detected on xylose-lysine-deoxycholate (XLD) agar (9) at a hospital lab. Notably, M001-2 and M964-3 were highly mucoid, morphologically typical of Gram-negative bacteria producing capsular polysaccharides (18). We confirmed every morphotype as Salmonella using matrix-assisted laser desorption/ionization time-of-flight mass spectrometry (MALDI-TOF MS) and observed each to be nontypeable even after several passages on blood agar, motility agar, or brain heart infusion (BHI) broth and after exposure to boiled water steam to remove any possible capsule. This contrasts with previous observations that mucoid forms from sero-untypeable Salmonella from patients with chest pleural effusion (19), iliopsoas abscess (20), or intramural thrombus (21) could be converted to concurrently isolated typeable nonmucoid forms after either culturing passages or heat steam exposure. However, the cause for expression of the two different forms of these Salmonella has not been previously reported.

**TABLE 1:** Heterogeneous Salmonella cell types identified from clinical specimens^*a*^

Specimen	Heterogeneous cell types	No.(s) of SNPs separating heterogenous cell genomes	ST	Genoserotype(s)
M211	Serotypes M211-2 (3,19:g,s,t:−), M211-5, and M211-9 (3,19:−:−)	3 for M211-2 and M211-5, 7 for M211-2 and M211-9	185	3:g,s,t:−
M378	Serotypes M378-3 (6,7:a:e,n,x), M378-4 (-:a:-), and M378-5 (-:a:e,n,x)	0 for M378-3 and M378-4, 3 for M378-3 and M378-5	1370	7:a:e,n,x for M378-3, −:a:e,n,x for the other two
M830	Morphotypes M830-1 (saw) and M830-2 (pas)	11	26	7:k:1,5
M736	Morphotypes M736-1 (rdar) and M736-2 (saw)	12	1561	13:i:1,2
M001	Morphotypes M001-1 (pdar) and M001-2 (saw/mucoid)	4	New type	IIIa 47:z4,z23:−
M557	Morphotypes M557-1 (bdar) and M557-2 (saw)	2	7750	IIIb 61:l,v:1,5,7
M964	Morphotypes M964-1 (ras) and M964-3 (saw/mucoid)	4	14	3:g,s,t:−

a *Salmonella* bacteria from a clinical specimen expresses heterogeneous serotypes (M211 and M378) or CR morphotypes (M830, M736, M001, M557, and M964). Serotype antigenic formulas and abbreviated morphotype descriptions are shown in parentheses in the table. Serotyping was performed by traditional agglutination testing using specific antisera. CR morphotypes were determined based on bacteria grown on Congo red agar (Fig. 1). SNP distances (≤12) were found between genomes of each group’s isolates (see Fig. S2 in the supplemental material). Each group was found to share a same sequence type (ST) or genoserotype except M378. The identified ST for M001 and genoserotype for M736 were not previously reported.

**FIG 2:**
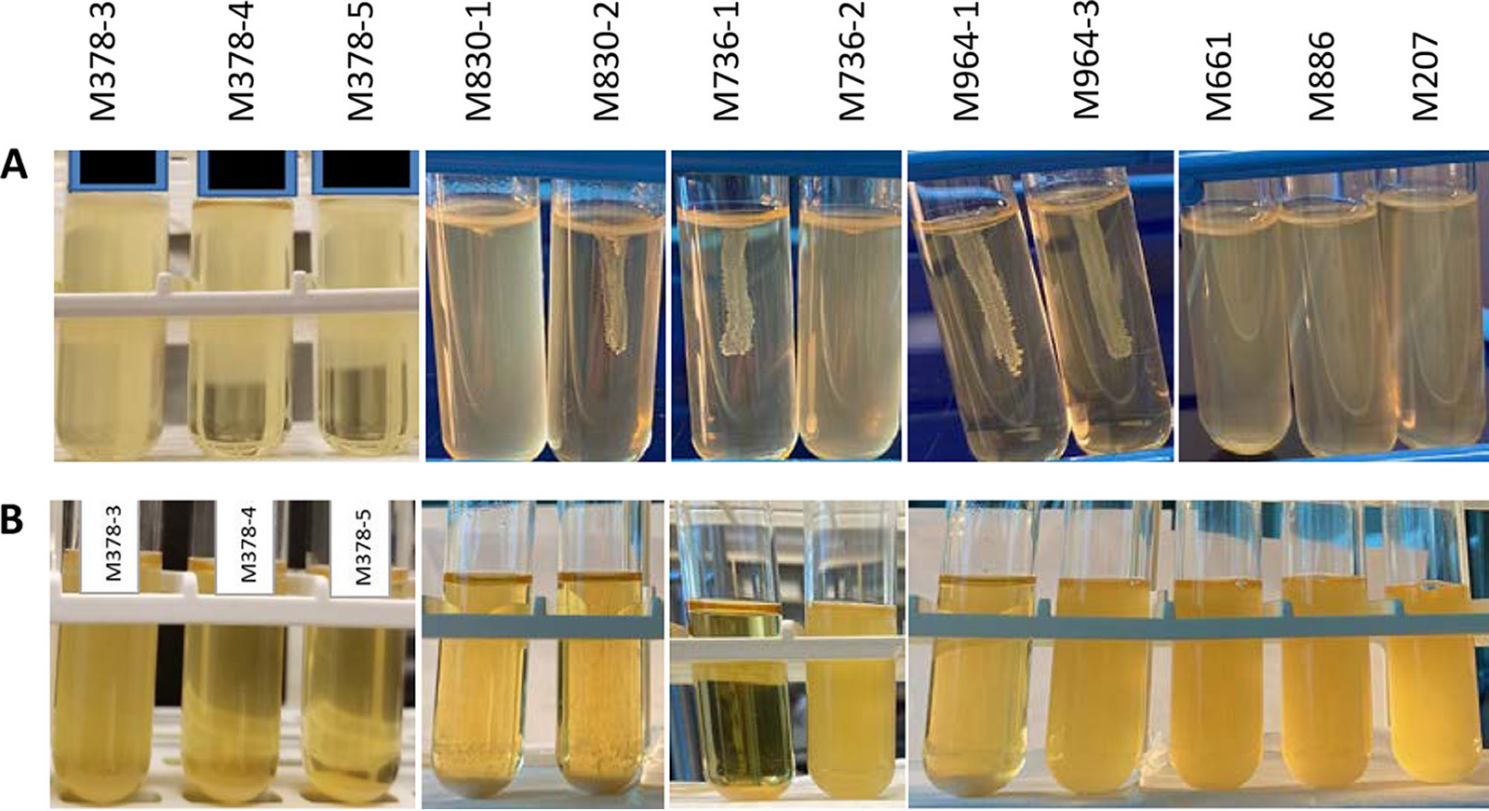
Growth difference by Salmonella of different serotypes and CR morphotypes from a clinical specimen. M378-3, compared to M378-4 and M378-5, is more motile on the motility test (A) and does not form cell precipitation in cultured BHI broth (B). Motility was also observed for M830-1 and M736-2, but not M830-2, M736-1, and the two M964 isolates. Different broth growth patterns were also noted between the isolate pair M736 and M964. Specimen isolates M661, M886, and M207 were collected earlier than the two M964 from the same patient in the course of their infections (Fig. 3A); these isolates were also noted to be motile in contrast to the two M964 isolates. Among the same patient’s five cultures, only M964-1 was found to form a cell precipitation in BHI broth. All cultures were incubated at 35°C for 18 h. No difference in motility and broth growth pattern was observed between each pair of M211, M001, and M557 groups, which are not presented here.

We also found other phenotype heterogeneity between isolates of a specimen. M378-3 was more motile than M378-4 and M378-5 on soft agar motility test and formed no cell precipitation in cultured BHI broth (Fig. 1), in which many motile bacteria were seen under microscope, in contrast to few motile organisms and many cell clumps in broths of the other two isolates (see Fig. S1 and its associated movie S1 and S2 files in the supplemental material). A difference was also noted in motility between two isolates from each of the M830 and M736 specimens and in broth growth patterns within M736 and M964 sets of isolates (Fig. 2). Biochemical reactions that are often used to identify Salmonella were also shown to vary within the following sets of isolates: (i) M830 for melibiose; (ii) M001 for malonate, inositol, and *o*-nitrophenyl-β-d-galactopyranoside (ONPG); and (iii) M736 for lysine, arginine, and ornithine (Table S1). A difference in susceptibility or MIC of antibiotics was also noted between M964-1 (≤1 μg/mL, sensitive) and M964-3 (2 μg/mL, intermediate) for imipenem and between M557-1 (≤4 μg/mL, sensitive) and M557-2 (>8 μg/mL, resistant) for tetracycline.

It is well recognized that heterogeneous bacteria can be grown from single-clone cells. The level of genetic and phenotypic diversity arising from genetically identical cells plays an important part in bacterial community properties and functions (22, 23). Examples of phenotypic diversity of Salmonella have been demonstrated with mouse model studies. S. *Typhimurium* was shown to divide into two subpopulations, with one rapidly proliferating subset expressing low levels of the invasion-associated type III secretion system genes and another slowly growing subset expressing high levels of the virulence genes (24) or shown to exhibit a different percentage of the flagellin-expressing subpopulation of the infected bacteria in animal Peyer’s patches versus spleen or mesenteric lymph nodes (25). To determine whether cultures isolated from the same patients but displaying heterogeneous phenotypes were genetically identical Salmonella, we performed WGS and a comparative analysis of their respective sequenced genomes. If genetic differences were seen, the analysis could help explain their polymorphism. In addition to SNPs, we looked for longer DNA sequence alterations in genes known previously for controlling the synthesis of bacterial cell membrane-associated molecules (e.g., LPS), as similar defects might have already been shown to correlate with our studied serotypes or morphotypes.

We found ≤12 SNPs separating each specimen set’s cell types, indicating heterogeneous cells evolving from the same progenitor, also suggested by their sharing of the same sequence type (ST) identified (Table 1). A new ST could be assigned to M001 because of its identified novel *aroC* allele that differentiated it from ST2023. Every group, except M378 (see below), was found to encode the same genoserotype (Table 1). The one for M736, 13:i:1,2, is not reflected in the current Kauffmann-Le Minor scheme (26, 27).

When searching for an underlying molecular mechanism for different serotypes by isolates within M211 and M378 specimens (Table 1), we observed that compared to M378-3, M378-4 and M378-5 had a 15-kb DNA region deleted from their genomes, which carries multiple O-factor synthesis genes such as *wcaJ*, *wzxC*, and *wcaK* (28) (Fig. S2). This deletion seems to be a cause for their phenotypes of no O-factor expression, as well as cell precipitation in culture broth and negative motility, as indicated previously that the loss of O-moiety (hydrophilic) from LPS resulted in a bacterial surface hydrophobicity increase followed by cell-to-cell clumping and concurrent flagellar apparatus assembly impairment on the cell surface membrane leading to defective motility for Salmonella (29). We did not, however, detect DNA sequence differences for different expression of H factors in the coding and upstream control (100-bp) regions of *fliC* gene (encoding the first phase of flagellin) and *fljBA* operon (encoding the second phase of flagellin) (30). It is recognized that besides flagellin-coding regions, more than 45 genes were shown to impact Salmonella flagellar formation at the bacterial cell surface, such as in regulating flagellin transport (31).

For the M830 group of isolates, we noted that an SNP-introducing stop codon present in both M830-1’s *csgD* and M830-2’s *waaK* genes could lead to truncation and subsequent function loss of either of these gene’s coded product (Fig. S2). Each such loss has been described before to impact multiple cellular events. Without CsgD (transcriptional regulator) in Salmonella, synthesis of curli, cellulose, or capsular polysaccharides is downregulated (32), while flagellar expression (33) and cell aggregation (34) are both promoted. These consequences are consistent with the “saw” morphotype, positive motility, and cell-clumping phenotype manifested by M830-1 (Fig. 1 and 2). Without the presence of WaaK as lipopolysaccharide α1,2-*N*-acetyl-glucosaminyltransferase, synthesis of the LPS core moiety is aborted, resulting in no O-antigen incorporation in LPS (35) and subsequent loss of the cell membrane integrity to support assembly of flagella anchored at the cell surface (36, 37). These effects seem reflected in related M830-2’s phenotypes (Fig. 2).

Compared to M736-2, the SNP in M736-1’s *ompR* gene can alter its coded transcription regulator’s structure (Q223H; Fig. S2) and subsequent DNA binding specificity, as shown previously with a same or adjacent amino acid sequence position replacement (38). The linked change to the EnvZ/OmpR two-component system will affect the downstream decarboxylation pathways of lysine, arginine, and ornithine by Salmonella when exposed to a high-acid environment (39, 40). Such hierarchy control by OmpR likely contributes to the M736-1’s phenotype being defective in all three decarboxylations (Table S1) despite encoding normal coding and upstream control DNA sequences for the decarboxylation process-associated genes of *cadA*, *cadB*, and *cadC* (lysine); *adiA*, *adiC*, and *adiY* (arginine); and *speF* (ornithine) (40), the same as M736-2 that expresses normal activity. Besides SNP, a longer DNA sequence change was noted between M736-1 and M736-2 within the region (*lpp*) coding for precursor lipoproteins (pre-LPPs), which are all composed of an LPP structure peptide preceded by a leader (signal) peptide sequence. The DNA region for M736-2 encodes three genes (*lpp1*, *lpp2*, and *lpp3*) in tandem, while present in M736-1 is a deleted version (in-frame) of an ~320-bp DNA segment between and extending partially into *lpp1* and *lpp2*. Therefore, M736-1, compared to M736-2, besides not producing a functional LPP1 structure peptide, encodes a recombinant pre-LPP composed of the same LPP2 structure peptide but preceded by LPP1’s signal peptide sequence (Fig. S2), which may undergo processing and trafficking inside the cell differently (41). As demonstrated previously in *S.* Typhimurium that an alteration of its *lpp* gene sequence, including the deletion of *lpp1*, can impair bacterial cell envelope structure and assembly of flagella (42, 43), we suggest that the similar change described above for the M736-1 *lpp* is a cause for its distinctive morphology and motility from M736-2 (Fig. 1 and 2).

It is likely that an SNP each present in M001-2’s *envZ* and *basR* (*pmrA*) genes, compared to M001-1, can change the enzymatic function of EnvZ (C339R) (44) and DNA binding activity of PmrA as transcription regulator (R117H) (45) (Fig. S2), respectively, and that the associated changes on EnvZ/OmpR and BasR/S (PmrA/B) two-component systems can cause expression of the mucoid morphotype as well as the unique biotype by M001-2, i.e., positive on inositol but negative on ONPG and malonate (Table S1). This biotype is exceptional to Salmonella enterica subspecies *arizonae* (IIIa) (27), to which M001-1 and M001-2 belong based on their genomes (Table 1).

As previously shown in Salmonella, the *ompR* gene can also regulate the synthesis of membrane-associated curli (46) and proteins (or porins) (47); we suggest that the M557-2’s *ompR*, with the SNP present making a different functional OmpR (M149T) (48) from M557-1 (Fig. S2), can likely cause M557-2’s “saw” morphotype with capsule (Fig. 1). The changed OmpR may also confer its resistance phenotype to tetracycline by altering bacterial cell surface structure, thereby reducing membrane drug permeability (49, 50). This is supported by the finding that none of the known resistance genes to tetracycline were detected from both isolates’ genomes. The SNP noted in the *rpoA* gene coding for RNA polymerase α subunit, known previously to control porin gene transcription initiation jointly with *ompR* (51), may also contribute to the phenotype difference.

**FIG 3:**
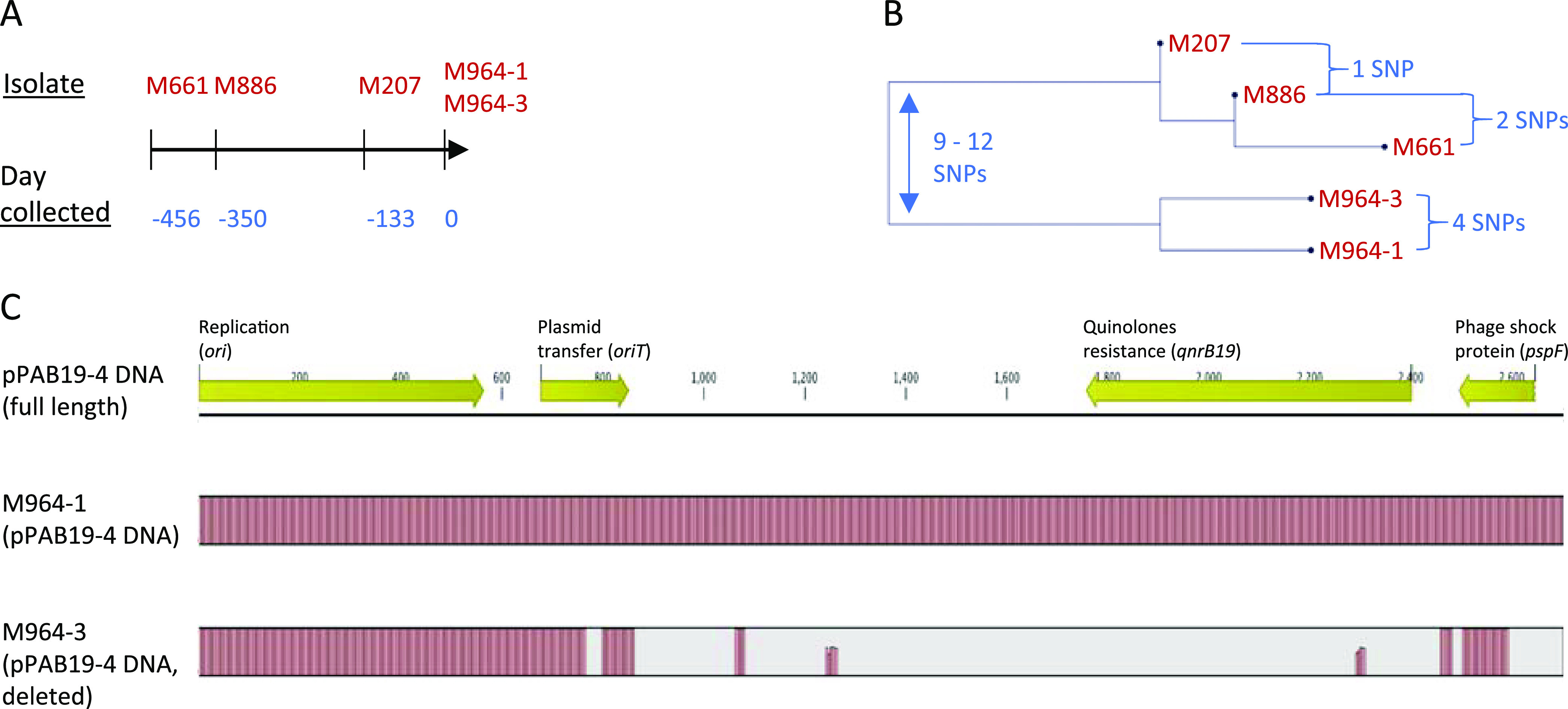
Characterization of Salmonella isolates collected from a patient over a course of persistent infection. (A) Prior to M964 collection, M661, M886, and M207 specimen isolates were collected at the indicated time. (B) SNP distances (≤12 SNPs) between sequenced genomes of all the isolates were determined to show their relatedness. (C) In genome analysis, M964-1 was found to carry the *qnrB19* gene harbored by a plasmid with >99.8% sequence similarity to pPAB19-4 (GenBank accession no. JN995611) (middle bar) in contrast to M964-3 carrying a deleted version (bottom bar) within the regions of plasmid transfer, resistance to quinolones, and phage shock protein. The alignment was completed using Qiagen CLC Genomics Workbench 10.1.1 software. M661, M886, and M207 were found to carry the same size of pPAB19-4 DNA as M964-1 and are not depicted here.

Salmonella cultures collected from a single patient with a long-term infection were also assessed; these include M964 (two isolates), M661, M886, and M207. The latter three were collected 456, 350, and 133 days earlier than M964, respectively (Fig. 3A). The five isolates were found to differ by ≤12 SNPs (Fig. 3B) and encode the same genoserotype of 3:g,s,t:−. The noted chronological order (Fig. 3A) reflects an immune evasion trend of gradual elimination of major surface (H and O) expression by Salmonella, isolates M661 or M886 (first and second collection) expressing both H and O (serotype 3,19:g,s,t:–), M207 expressing only H (serotype –:g,s,t:–) (third collection), and M964 (last collection) expressing neither. The negative expression of H antigen and defective motility by both M964 isolates (Fig. 2) is because of the presence of an SNP introducing premature stop codon in their *fliF* gene coding for flagellar M-ring motor protein essential for assembly of functional flagella (52) (Fig. S2). Between the two M964 isolates, SNPs were found associated with null (*rcsC*) and nonsynonymous (*rcsC* and *rcsB*) changes in the individual gene-translated peptides (Fig. S2). Alterations in the *rcsC*/*rcsB*-associated two-component system have been reported to impact multiple cellular events, including the addition of repeated O-antigen units to the LPS core and formation of capsule polysaccharide (53); thus, it can explain the heterogeneous motility and CR morphotype between the two M964 isolates (Fig. 1 and 2). Of note, Salmonella mutants constructed with defects in the two-component system have been described to result in a prolonged infection in animals (54, 55). In comparing molecular determinants for antibiotic resistance between heterogenous cell genomes, we observed that the *qnrB19* gene, conferring plasmid-mediated (56) resistance to quinolones (e.g., fluoroquinolones), was present in M964-1 and the three isolates collected at earlier time points, but not in M964-3. The gene was found to be harbored by pPAB19-4 and was deleted from the plasmid only in M964-3 (Fig. 3C).

In summary, we showed here Salmonella subpopulations coexist in the patient specimens collected at a single disease point, which contrasts with previous work on the isolation of single different populations at different time points over the course of an infection (13–15). The subpopulations were observed to express different phenotypes related to cell surface antigen expression, motility, biofilm formation, biochemical metabolism, and antibiotic resistance. Aside from the same specimen, heterogeneity was also observed between Salmonella isolated from blood and stool specimens of the same patient in expressing an H antigen factor (F. Xu, unpublished data). There is likely additional unfound genetic heterogeneity between our studied pairs, as their SNPs in core genomes were only considered when they were being mapped to the reference genome. The method was chosen to enable determination of genetic relatedness. The generated results do not capture variation in genomic regions that are not in the reference genome. Also, we did not perform a comprehensive search for mutations of longer DNA sequences than SNPs. Some of them may likely be in genes playing a regulatory or indirect role in the phenotypes that we described. So far, our study suggests that, like in animal models, Salmonella adopts a dynamic adaption strategy by creating subpopulations of different functions, notably in modifying two-component regulatory systems, to achieve, as a community, prolonged infection in humans. Future cell-based and animal model studies comparing the impact of individual subpopulations and their combinations can support this notion. Our study also provides clinical and public health insights into future laboratory diagnosis of salmonellosis. The current practice for testing clinical isolates from a single colony pick or single cell type, even if it is the predominant population, may fail to control residual salmonellosis caused by an undiagnosed subpopulation in a patient who could continuously shed pathogens. This is a particular concern for patients receiving antibiotic therapy for their Salmonella infections. As heterogenous Salmonella subpopulations have also been found in environmental samples, such as oregano (57), tomato (58), and pork (Y. Gebremichael and F. Xu, unpublished data), future surveillance and epidemiologic investigations, including source tracking and characterization of antibiotic resistance, should include testing and analysis of multiple Salmonella subpopulations from clinical and environmental sources.

## MATERIALS AND METHODS

### Clinical Salmonella specimens.

Clinical cultures were initially identified as Salmonella by hospital laboratories in California and submitted through local public health laboratories to the California Department of Public Health, Microbial Diseases Laboratory.

### Salmonella culture and serotyping.

When investigating the possible presence of heterogenous serotypes expressed by different Salmonella in a specimen, it was first plated on brain heart infusion (BHI; Difco) agar to provide well-separated colony isolates. Ten of them were then randomly selected for a serotype identification screening assay. One or two isolates representing a unique serotype were randomly selected from each screened pool for further phenotypic and genetic characterizations. Serotyping was performed by agglutination to detect the presence of O and H antigen factors using specific antisera (59). The antisera were prepared by our laboratory following the guidelines from the WHO Centre for Reference and Research on Salmonella at the Institute Pasteur, Paris, France. Serotypes were assigned using the Kauffmann-White-Le Minor scheme (27, 28). Salmonella isolates were also grown in BHI broth or inoculated into motility GI medium (3% agar; Difco) for examination of cell growth and motility after 18 h incubation at 35°C.

### Congo red agar plating assay.

Salmonella isolates were assessed for differential colonial morphology, which can be used to predict bacterial biofilm formation traits (16). The base medium, derived from Luria-Bertani (LB) broth without salt, includes yeast extract (5 g/L; Becton, Dickinson) and tryptone (10 g/L; Becton, Dickinson). The final agar medium was made from the base medium supplemented with Congo red (40 mg/L; Sigma), Coomassie brilliant blue G-250 (20 mg/L; Sigma), and agar powder (2 g/L; Becton, Dickinson). Bacterial colonial morphology was examined after 48 h incubation at 35°C.

### Microscopic examination.

After growth overnight at 35°C in BHI broth culture, Salmonella cell motility and aggregation were examined using 5 μL of cell suspension placed onto a coverslip. The coverslip was inverted, gently pressed against a two-ring etched slide, and sealed with petrolatum wax. Live cells were viewed with a Leica DM5500 B microscope using phase-contrast at ×1,000 magnification and recorded using a Leica DFC550 camera.

### Antibiotic sensitivity and biochemical metabolic activity measurement.

MicroScan Neg/Urine Combo panel type 73 (Beckman) was used to determine antibiotic susceptibility and biochemical metabolic activity for bacterial cultures. Panels containing biochemicals and antibiotics were incubated for 18 h and read on the MicroScan WalkAway SI system. Results were analyzed with LabPro software. Breakpoint values for sensitive, intermediate, and resistant to an antibiotic were referenced from current Clinical and Laboratory Standards Institute (CLSI) instructions as stated in the product manual (60). Biochemical activity was also measured with test tube media for lysine, arginine, ornithine, H_2_S, *o*-nitrophenyl-β-d-galactopyranoside (ONPG), melibiose, malonate, and inositol (Thermo Fisher).

### WGS.

Salmonella genomic DNA was extracted from isolated bacterial colony cells using the DNeasy blood and tissue kit (Qiagen). The DNA purity was measured using a NanoDrop 2000 UV-visible (UV-Vis) spectrophotometer (Thermo Fisher), and the concentration was determined using the Qubit double-stranded DNA (dsDNA) broad-range (BR) assay kit (Thermo Fisher). The DNA library was dsDNA high-sensitivity (HS) assay kit (Thermo Fisher). The library size distribution was analyzed on a 2100 BioAnalyzer instrument using the high-sensitivity DNA kit (Agilent). Sequencing was performed on a MiSeq sequencer using the Illumina MiSeq reagent kit (2 × 250 cycles) and PhiX DNA as control. Sequence reads generated gave the genome depth coverage of ≥60×.

### Bioinformatic analyses.

Paired-end reads were quality trimmed at the threshold of Q30 and then mapped to the reference genome using Qiagen CLC Genomic Workbench 10.1.1 (CLCbio; Qiagen). The reference genome used for SNP-based phylogenetic analysis is a complete genome and closely related to the strains in the analysis. The selection for the reference genome was made by using the KmerFinder 3.2 webtool by the Center for Genomic Epidemiology (CGE; https://www.genomicepidemiology.org/), which produces top hits for genomes homologous to the strains. The analyzed genome sequences were masked for present phages and repetitive sequences that showed up from the reference genome after analysis using the PHAST webtool (http://phast.wishartlab.com/). After mapping, the BAM files generated were processed using a series of software suites. A customized shell script (https://github.com/ritumukh/MiSeq_CDPH) was created to automate the subsequent steps that included (i) SNP calling in both coding and noncoding regions using SAMtools mpileup (v.1.2) (61), (ii) converting into VCF matrix using bcftools (v.0.1.19; http://samtools.github.io/bcftools/); (iii) variant parsing using vcftools (v.0.1.12b) to include only high-quality SNPs (hqSNPs) with ≥30× genome coverage plus quality score >200, excluding any DNA insertion and deletion (indels) or heterozygote calls; and (iv) converting the SNP matrix into a FASTA alignment file compatible with CLC for generating an unrooted phylogenetic tree (for specimen isolates from the long-term diseased patient). The SNP list is filtered to remove any indels and heterozygote calls. No other filtration criteria or tools are used. A maximum-likelihood tree was created from the called high-quality SNPs (hqSNPs) following the Jukes-Cantor nucleotide substitution model; bootstrapping included 100 replicates. The *de novo* assembly of a genome was generated using CLC. The identified SNPs were confirmed with alignment of *de novo* assemblies to each other and to reference genome using the CLC Whole Genome Alignment tool (https://digitalinsights.qiagen.com/plugins/whole-genome-alignment/). The bacterial species identification was performed using the KmerFinder 3.2 webtool. *In silico* MLST (sequence type) results showing sequence variations (alleles) in seven target housekeeping genes were determined using the MLST 2.0 webtool by CGE. Antibiotic resistance genes present in the sequenced genomes were identified through matching for known resistance determinants using the ResFinder 3.2 webtool by CGE (62). Predicted serotypes (genoserotype) based on Salmonella genomes were achieved using SeqSero 1.2 webtool (63). For other genome analyses, including a search for sequence deletion, CLC was used.

### Ethics statement.

The study presented with the use of highly deidentified human specimens was under review and clearance by the California Department of Public Health and adheres to public laws.

### Data availability.

Raw sequencing reads are deposited in NCBI BioProject under accession no. PRJNA820770. Genome assemblies are deposited in GenBank with accession numbers JAOPIE000000000 (M964-3), JAOPIF000000000 (M964-1), JAOPIG000000000 (M886), JAOPIH000000000 (M830-2), JAOPII000000000 (M830-1), JAOPIJ000000000 (M736-2), JAOPIK000000000 (M736-1), JAOPIL000000000 (M661), JAOPIM000000000 (M557-2), JAOPIN000000000 (M557-1), JAOPIO000000000 (M378-5), JAOPIP000000000 (M378-4), JAOPIQ000000000 (M378-3), JAOPIR000000000 (M211-9), JAOPIS000000000 (M211-5), JAOPIT000000000 (M211-2), JAOPIU000000000 (M207), JAOPIV000000000 (M001-2), and JAOPIW000000000 (M001-1).
