# Supplementary material for: Salmonella Subpopulations Identified from Human Specimens Express Heterogenous Phenotypes That Are Relevant to Clinical Diagnosis
Source: Microbiol Spectr. 2022 Dec 12;11(1):e01679-22. doi: 10.1128/spectrum.01679-22 (PMC9927314; doi:10.1128/spectrum.01679-22)
Supplement: Supplemental file 2 — Fig. S1 and S2. Download spectrum.01679-22-s0002.pdf, PDF file, 0.3 MB [file spectrum.01679-22-s0002.pdf]

FIG S1 Live microscopic imageries of *Salmonella* cells that display heterogenous serotypes from specimen M378. After the cells were cultured in BHI broth at 35°C for 18 hours, a culture suspension was examined using a Leica DM5500B microscope under x1000 magnification and phase-contrast light view setting. The M378-3 culture was observed to have highly motile cells and no cell aggregation (A), in contrast to M378-4 culture which contains few motile bacteria and many cell clumps (B). A scale bar for the imageries is provided. The M378-5's imagery is similar to M378-4's and not presented here. The live imageries are presented in "movie files".

A. M378-3

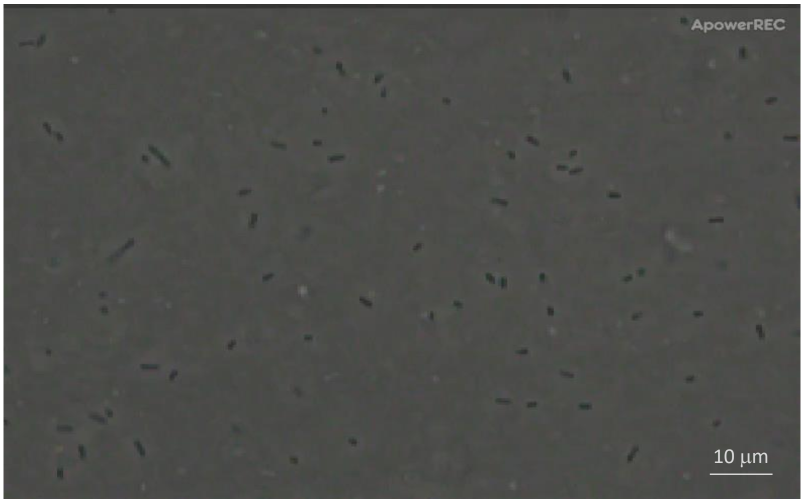

B. M378-4

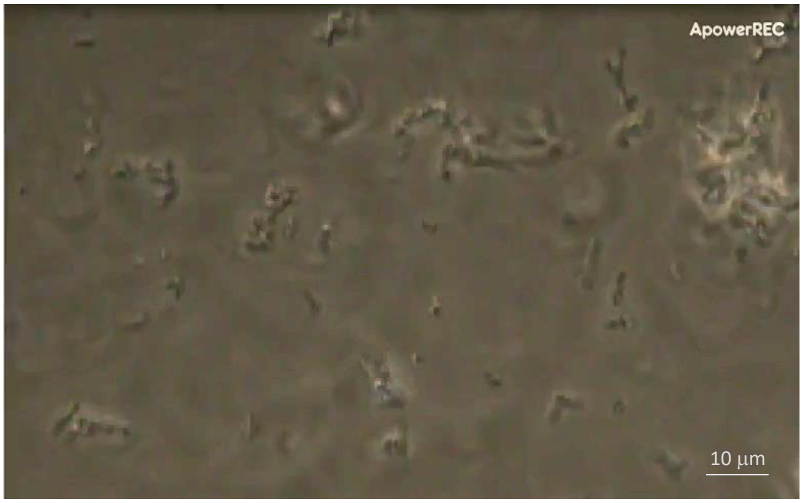

FIG S2 SNPs and their linked regions in genomes of heterogenous *Salmonella* isolates from clinical specimens. SNPs were identified on assembled bacterial genome with comparing to reference genome. The indicated SNP positions are based on individual reference genome sequences used (NCBI accession ID provided). SNP-linked changes in specific genes and their coded products or in intergenic DNA regions are shown. The identified SNPs are not within the consensus bacterial promoter sequence (or TATA box); and, if present upstream from the start codon of a gene, are >150 bp away. Longer DNA sequence changes than single nucleotide that were identified to alter the O antigen (for M378) and lipoprotein (for M736) coding regions are also described here.

A. M211 specimen[a]

SNP	Position in reference NZ_CP016837.1	Sequence change in heterogenous isolates			Changed DNA	Changed gene product
		M211-2	M211-5	M211-9		
1	1,427,464			G to A	*guaB*	IMP dehydrogenase (D50N)
2	1,711,921			C to T	*nudI*	nucleoside triphosphatase (I76I)
3	1,934,827	C to T			*rfbB*	dTDP-glucose 4,6-dehydratase (A348V)
4	3,789,443	G to A			*rpsB*	30S ribosomal protein S2 (A12V)
5	545,819			G to A	Intergenic region	
6	3,805,150			G to A	Intergenic region	
7	4,048,572	G to A			Intergenic region	

[a]It remains to be further studied about whether the above SNP-associated genes and intergenic regions can cause different expression of H antigen factors between M211-2 and the other two isolates.

B. M378 specimen[b]

SNP	Position in reference NZ_CP030231.1	Sequence change in heterogenous isolates			Changed DNA	Changed gene product
		M378-3	M378-4	M378-5		
1	612,453			C to A	*siaT*	Sialic acid TRAP transporter permease protein (A343A)
2	2,273,273			C to T	*hpaA*	4-hydroxyphenylacetate catabolism regulatory protein, Exoenzyme S synthesis regulatory protein (A271V)
3	4,556,512			T to G	*rbsR*	ribose operon transcriptional repressor (L51Stop)

[b]It remains to be further studied about whether the above SNP-associated genes are responsible for different expression of H antigen factors between the M378 isolates. However, a deletion of 14,701 bp (reference genome positions: 3,261,820 to 3,276,521) containing O antigen synthesis genes: *wcaJ, wzxC, wcaK, wcaL, wcaM, galF* found in M378-4 and M378-5, apparently can cause their negative O serotype (28).

A. M830 specimen[c]

SNP	Position in reference NZ_CP040699	Sequence change in heterogenous isolates		Changed DNA	Changed gene product
		M830-1	M830-2		
1	131,039		C to T	*waaK*	lipopolysaccharide N-acetylglucosaminyltransferase (Q29Stop)
2	545,782		G to A	*gltB*	glutamate synthase [NADPH] large chain precursor (E871K)
3	2,707,696	C to T		*csgD*	CsgBAC operon transcriptional regulatory protein (Q205Stop)
4	3,016,095		C to G	*ybhS*	Inner membrane transport permease (D127E)
5	3,158,395		G to A	*rihA*	pyrimidine-specific ribonucleoside hydrolase (E252K)
6	3,442,840		A to T	*adrA*	Putative diguanylate cyclase (Q96L)

7	3,838,642	C to T		*oadA*	sodium-extruding oxaloacetate decarboxylase subunit alpha (A234A)
8	4,155,632		A to G	*hfq*	RNA-binding protein (T61A)
9	4,373,736	C to A		*hupA*	DNA-binding protein HU-alpha (A24D)
10	227,543	T to C		Intergenic region	
11	2,418,663	G to A		Intergenic region	

[c]SNPs introducing "Stop" codons that lead to the changed *waaK* and *csgD* gene products are indicated, which are apparently a cause for the studied different phenotypes between the two M830 isolates (see text for details). It remains to be further studied about whether changes in other indicated DNA have a similar effect.

D. M736 specimen[d]

SNP	Position in reference NZ_CP022116	Sequence change in heterogenous isolates		Changed DNA	Changed gene product
		M736-1	M736-2		
1	383,105	G to T		*ompR*	Transcriptional regulatory protein (Q223H)

[d]Apart from SNP, different *lpp* regions were found between M736-1 and M736-2 genomes (see below diagram). Each of the *lpp* genes encodes a lipoprotein structural peptide preceded by a leader (or signal) peptide sequence separated by cysteine residue as indicated. The dash-line representing about a 320-bp segment between and partially overlapping with *lpp1* and *lpp2* (in M736-2) is in-frame deleted from M736-1, shown below in both diagram and DNA sequence alignment illustration with corresponding regions of same color labelling. Therefore, M736-1 does not encode a functional LPP1 structure peptide but encodes a recombinant pre-LPP composed of LPP2's structure peptide (green) preceded by LPP1's signal peptide sequence (purple). The M736-2 DNA segment in below diagram is between positions of 2192000-2193000 as to the reference genome.

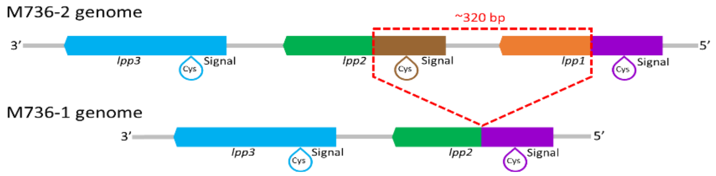


```
M736-2     1  ATGAATCGTACTAAACTGGTACTGGGCGCGGTAATCCTGGGTTCTACTCTGCTGGCTGGT  60
             ||||||||||||||||||||||||||||||||||||||||||||||||||||||||||||
M736-1     1  ATGAATCGTACTAAACTGGTACTGGGCGCGGTAATCCTGGGTTCTACTCTGCTGGCTGGT  60

M736-2    61  TGCTCCAGCAACGCTAAAATCGATCAGCTGTCTTCTGACGTTCAGACTCTGAACGCTAAA 120
              |||||||||||||||||||||||||||||||||||||||||||||
M736-1    61  TGCTCCAGCAACGCTAAAATCGATCAGCTGTCTTCTGACGTTCAG--------------- 105

M736-2   121  GTTGACCAGCTGAGCAACGACGTGAACGCAATGCGTTCCGACGTTCAGGCTGCTAAAGAC 180

M736-1        ------------------------------------------------------------

M736-2   181  GACGCAGCTCGCGCTAACCAGCGTCTGGACAACCAGGCTACTAAATACCGTAAGTAATAG 240

M736-1        ------------------------------------------------------------

M736-2   241  TACCTGCGTAATAAAAATGGCGCACATCGTGCGCCATTTTTTTTACCCACATAACCACAC 300

M736-1        ------------------------------------------------------------

M736-2   301  AAAGTATAATGTTATTGTTATGAACCGTACTAACCAGCTGATCTTAGGTGCAGTAGTTCT 360

M736-1        ------------------------------------------------------------

M736-2   361  GGGTTCCACGTTACTGGCAGGTTGTTCAAGCAACGCTAAAATCGATCAGTTGTCTTCCGA 420

M736-1        ------------------------------------------------------------

M736-2   421  TGTACAGACGCTGAGCGCTAAAGTTGATCAACTGAGTAATGACGTAAATGCAATGCGTTC 480
                     |||||||||||||||||||||||||||||||||||||||||||||||||||||
M736-1   106  -------ACGCTGAGCGCTAAAGTTGATCAACTGAGTAATGACGTAAATGCAATGCGTTC 158

M736-2   481  CGACGTTCAGGCTGCTAAAGACGATGCTGCTCGCGCGAACCAGCGTCTGGACAACAAAGT 540
              ||||||||||||||||||||||||||||||||||||||||||||||||||||||||||||
M736-1   159  CGACGTTCAGGCTGCTAAAGACGATGCTGCTCGCGCGAACCAGCGTCTGGACAACAAAGT 218
```

```
M736-2     541 AGCATCCCACGTCCGTAAGTAA 562
               ||||||||||||||||||||||
M736-1     219 AGCATCCCACGTCCGTAAGTAA 240
```

E. M001 specimen[e]

SNP	Position in reference NZ_CP022116	Sequence change in heterogenous isolates		Changed DNA	Changed gene product
		M001-1	M001-2		
1	384,167		C to T	*envZ*	Osmolarity sensor protein (R339C)
2	3,996,369		T to C	*arcA*	Aerobic respiration control protein (I90T)
3	4,391,295	G to A		*basR*	Transcriptional regulatory protein (R117H)
4	2,592,769	C to A		Intergenic region	

[e]The SNP-associated changes in *envZ* and *basR* (*pmrA*) genes can result in different CR morphotypes by the two isolates (see text for details). It remains to be further studied about whether changes in other indicated DNA can have a similar effect.

F. M557 specimen[f]

SNP	Position in reference NZ_CP034074	Sequence change in heterogenous isolates		Changed DNA	Changed gene product
		M557-1	M557-2		
1	337,995		C to T	*ompR*	Transcriptional regulatory protein (T149M)
2	421,495	C to T		*rpoA*	DNA-directed RNA polymerase subunit alpha (R310C)

[f]SNP-linked changes in *ompR* and *rpoA* genes can likely change their encoded protein function as a global gene expression regulator, leading to the studied phenotype changes between the two isolates (see text for details).

G. M964 specimen (heterogenous isolates: M964-1 and M964-3) and 3 earlier time collected specimen isolates (M661, M886, M207) from a same patient[g]

SNP	Position in reference NZ_CP039270.1	Sequence change in isolate					Changed DNA	Changed gene product
		M661	M886	M207	M964-1	M964-3		
1	1,767,990				G to A		*rcsC*	two-component system sensor histidine kinase (W127Stop)
2	1,770,240				A to G	A to G	*rcsC*	two-component system sensor histidine kinase (N749S)
3	1,771,042				T to C		*rcsB*	transcriptional regulator (F17S)
4	1,771,161					C to T	*rcsB*	transcriptional regulator (L57F)
5	2,686,129				T to C	T to C	*topA*	type I DNA topoisomerase (I525T)
6	2,929,659				C to T	C to T	*fliF*	flagellar M-ring protein (Q319Stop)
7	3,479,355				C to T	C to T	*ramA*	RamA family antibiotic efflux transcriptional regulator (T70I)
8	4,114,977				C to T	C to T	*chiA*	Chitinase (A360V)
9	4,209,963			G to A	G to A	G to A	*iadA*	beta-aspartyl-peptidase (M132I)
10	4,512,054				T to C	T to C	*gfcC*	capsule biosynthesis GfcC family protein (V147A)
11	4,691,412		G to A	G to A	G to A	G to A	*rhaA*	L-rhamnose isomerase (L325L)

12	3,874,005	T to G					Intergenic region	
13	4,547,498				G to T	G to T	Intergenic region	

[g]The SNP-linked changes in genes of *fliF*, *rcsB*, and *rcsC* are apparently a cause for variation in the studied phenotypes by the isolates (see text for details). It remains to be further studied about whether mutations in other indicated DNA regions have a similar effect.
